# Walking away from back pain: one step at a time – a community-based randomised controlled trial

**DOI:** 10.1186/s12889-015-1496-9

**Published:** 2015-02-13

**Authors:** Stephan Milosavljevic, Lynne Clay, Brenna Bath, Catherine Trask, Erika Penz, Sam Stewart, Paul Hendrick, G David Baxter, Deirdre A Hurley, Suzanne M McDonough

**Affiliations:** University of Saskatchewan, School of Physical Therapy, 1121 College Drive, Saskatoon, SK S7N 0W3 Canada; University of Saskatchewan, Canadian Centre for Health and Safety in Agriculture, 104 Clinic Place, PO Box 23, Saskatoon, SK S7N 2Z4 Canada; Respirology, Critical Care and Sleep Medicine, Department of Medicine, University of Saskatchewan and Saskatoon Health Region, Royal University Hospital, 103 Hospital Drive, Saskatoon, SK S7N 0W8 Canada; Division of Physiotherapy and Rehabilitation Sciences, School of Health Sciences, The University of Nottingham, B90, Clinical Sciences Building, City Hospital Campus, Nottingham, NG5 1PB UK; Centre for Health, Activity, and Rehabilitation Research, University of Otago, PO Box 56, Dunedin, 9054 New Zealand; School of Public Health, Physiotherapy and Population Science, UCD Health Sciences Centre, University College Dublin, Belfield, Dublin 4, Ireland; School of Health Sciences, Institute of Nursing and Health Research, University of Ulster, Shore Road, Belfast, BT37 0QB UK

**Keywords:** Chronic low back pain, Physical activity, Pedometer-driven walking

## Abstract

**Background:**

Low back pain is highly prevalent and a significant public health burden in Western society. Feasibility studies suggest personalised pedometer-driven walking is an acceptable and effective motivating tool in the management of chronic low back pain (CLBP ≥ 12 weeks). The proposed study will investigate pedometer-driven walking as a low cost, easily accessible, and sustainable means of physical activity to improve disability and clinical outcomes for people with CLBP in Saskatchewan, Canada.

**Methods/design:**

A fully-powered single-blinded randomised controlled trial will compare back care advice and education with back care advice and education followed by a 12-week pedometer-driven walking programme in adults with CLBP. Adults with self-reported CLBP will be recruited from the community and screened for elibility. Two-hundred participants will be randomly allocated to one of two intervention groups. All participants will receive a single back care advice and education session with a physiotherapist. Participants in the walking group will also receive a physiotherapist-facilitated pedometer based walking programme. The physiotherapist will facilitate the participant to monitor and progress the walking programme, by phone, on a weekly basis over 10 weeks following two face-to-face sessions. Outcome measures of self-reported disability, physical activity, participants’ low back pain beliefs/perceptions, quality of life and direct/indirect cost estimates will be gathered at baseline, three months, six months, and 12 months by a different physiotherapist blinded to group allocation. Following intervention, focus groups will be used to explore participants’ thoughts and experiences of pedometer-driven walking as a management tool for CLBP.

**Discussion:**

This paper describes the design of a community-based RCT to determine the effectiveness of a pedometer-driven walking programme in the management of CLBP.

**Trial registration:**

United States National Institutes of Health Clinical Trails registry (http://ClinicalTrails.gov/) No. NCT02284958. Registered on 27^th^ October 2014).

## Background

Chronic low back pain (CLBP) – back pain present for greater than three months [1] – is a prevalent, disabling, and costly musculoskeletal disorder in need of effective, efficient, and accessible interventions [2]. Low back pain (LBP) is a substantial public health burden in western society [3]. In Canada, the cost of medical expenditure for low back pain is estimated at up to $12 billion annually with additional costs linked to loss in worker productivity, lost work time, and associated disability payments [4,5]. Although research describes an episode of acute LBP as predominantly self-limiting [6], it is also known to be the most significant predictor of CLBP [7,8]. Four out of five Canadians will experience at least one episode of LBP at some point in their life and one in five adult Canadians will report the presence of CLBP at any given time [9].

CLBP is also significantly associated with a range of psychological and physical comorbidities and lower socioeconomic status [10]. Being overweight or obese increases the risk of LBP, and has the strongest association with seeking care for CLBP [11]. In contrast, reduction in weight improves outcomes for patients with musculoskeletal disorders [12]. Although the relationship between weight and physical activity is mediated by a variety of factors, the modern Canadian lifestyle is more sedentary with many people working in seated positions and a greater use of vehicles for transportation [13]. It has been posited that work pressure and other priorities create a perception of less time to undertake fundamental physical activities, such as walking, that are known to offer substantial health benefits [13]. A recent systematic review and meta-analysis shows the positive effect of walking, a basic human activity, on chronic musculoskeletal pain and self-reported disability [14].

Much attention has been devoted to interventions and strategies for managing CLBP; however, effective management remains elusive [15,16]. Interestingly, although walking is recognised as a fundamental human activity, there is limited literature on its use as an intervention strategy for CLBP [14,17-21]. Further research is required to determine the effectiveness of walking programmes as an intervention for CLBP and, subsequently, to develop guidelines on how to implement such programmes. Encouraging people with CLBP to increase physical activity through walking, however, can be problematic due to fear of pain exacerbation [6]. A systematic review suggests there is strong evidence to using pedometer-based walking interventions to increase physical activity and enhance quality of life in people with musculoskeletal disorders [22]. Researchers in the United Kingdom have recently completed a feasibility study of a community-based, pedometer-driven clinician guided walking programme for CLBP and found high levels of patient satisfaction and adherence, increased walking, improved physical function, reduced disability, and reduced pain, all demonstrating support for more definitive clinical trials [21].

Saskatchewan is a Canadian province with approximately 30% of the population residing in rural communities in which agriculture is a key industry [23]. Prevalence data for LBP in Saskatchewan shows point and lifetime prevalence at 28% and 84% and low to high disability CLBP ranging from 49% to 11% respectively [24]. People living in rural and remote areas of Saskatchewan are more likely to be afflicted with low back disorders [10,25]. Farmers and rural workers are much more prone to LBP [26]. The physical demands of farming combined with the very long work hours during planting and harvesting [27] and physical exposures such as whole body vibration and heavy lifting present a uniquely vulnerable scenario [28,29]. Back problems impact negatively on farming productivity; influencing work activity and reducing farm income [30]. Although farming is considered to be a physically active occupation, reduced activity has been reported in those with chronic health conditions [31].

### Aim and objectives

The proposed study will investigate pedometer-driven walking as a low-cost, easily accessible, and sustainable means of physical activity to improve disability and clinical outcomes for people with CLBP in Saskatchewan, Canada. Our hypothesis is that individualised advice and education followed by a pedometer-driven walking programme will be a more clinically and cost-effective option for the management of CLBP compared to advice and education alone. Results from this study will inform future exercise-based strategies for larger comparative international clinical trials aimed at reducing disability in those suffering from CLBP. The study’s objectives are fourfold:

1. To determine perceived levels of disability and baseline levels of walking activity in a sample of Saskatchewan residents with CLBP.
2. To determine the uptake and adherence to a pedometer-driven walking programme for people with CLBP.
3. To test the difference in clinical and cost-effectiveness of a walking programme to improve outcomes for CLBP compared to an individualised back care education package.
4. To test the feasibility of a walking programme in a sub-sample of rural Saskatchewan farmers/agricultural workers. Specifically, we will compare recruitment and adherence rates of farmers relative to non-farmers in light of the unique work demands, work and living environment in the rural community.

## Methods/design

### Study design

A single-blinded randomised-controlled trial (RCT) will be used to evaluate the effectiveness of a 12-week individualised pedometer-driven walking programme in people self-identifying with CLBP. Participants will be randomised on a 1:2 basis into a “Back care advice and education” group or a “Back care advice and education plus pedometer-driven walking” group. Outcome measures will be collected at baseline, three months (or post-intervention), six months, and 12 months. The structure and reporting of this trial will be guided by the CONSORT statement for clinical trials [32].

### Ethical approval

The study has been granted approval by the University of Saskatchewan Biomedical Ethics Board (#14-218) and registered with the United States National Institutes of Health Clinical Trails registry (https://clinicaltrials.gov No. NCT02284958). Written informed consent from all participants will be required prior to entering the study.

### Description and selection criteria of participants

A pragmatic, community-based approach will be used to recruit adults with CLBP residing in urban and rural communities within the boundaries of the Saskatoon Health Region, Saskatchewan, Canada. A wide variety of recruitment strategies such as posters, fliers, clinical and public notice boards, newspapers, and electronic bulletin boards will be used to ensure a mixed group of people representative of the CLBP population. People under active treatment by a health professional will be eligible to take part, as will those currently receiving no clinical intervention. Programme intake will be ongoing as participants are recruited from the community over time. We expect to complete full recruitment by 15 months and complete the full 12-month follow up by 27 months.

In order to address the higher prevalence and risk for CLBP among the rural farming workforce, we will specifically recruit a minimum of 20 participants with CLBP who live in the rural community and work on farms geographically located within the Saskatoon Health Region. These 20 participants will be part of the full recruitment strategy for random allocation and, thus, will also provide a subgroup for investigation of intervention feasibility within this unique vulnerable population.

### Screening

Study inclusion and exclusion criteria are presented in Table 1. Potential participants will be screened for eligibility over three stages: 1) by a research assistant over the phone; 2) by a physiotherapist following clinical examination and; 3) following a pedometer trial.

**Table 1:** **Inclusion and exclusion criteria**

**Inclusion criteria**	**Exclusion criteria**
Males and females aged 18 years or over	Any spinal surgery in the past 12 months
LBP (i.e. between the 12th costal margin and gluteal fold with or without associated leg pain) persisting for a minimum of three months	Evidence of nerve root, spinal cord, or cauda equina compression assessed by a physiotherapist
Physically able to participate in a walking programme as determined by the PAR-Q*	Current lower extremity musculoskeletal injury, cardiorespiratory or other medical condition that may be a contraindication to increasing physical activity levels
Average daily walking is fewer than 7500 steps as determined by a one week pedometer trial	History of serious psychological or psychiatric illness
	Current pregnancy
	Average daily walking is greater than 7500 steps as determined by a one week pedometer trial

*PAR-Q – Physical Activity readiness Questionnaire (Shephard 1988). If a participant answers yes to one or more questions on the PAR-Q, they will be advised to consult their primary healthcare provider to obtain physical clearance prior to confirming study participation.

#### Pedometer trial

Individuals deemed clinically suitable for the study will be invited to wear a sealed pedometer for seven days in order to establish a baseline measurement of steps [20]. Although there has been little research on step count thresholds for inclusion in a CLBP study, previous research and review by Tudor-Locke and colleagues [33,34] has identified ~7,500 steps as a baseline cut-off for interventions relative to health improvement. Therefore, only potential participants who walk less than an average 7,500 steps per day, over the seven day trial, will be invited to take part in the full study.

### Outcome measures

All eligible participants will meet with a blinded assessor (a different physiotherapist from the research team) to complete a battery of questionnaires. Measures will be taken at four time points; baseline, three months (or, for the walking group, immediately following intervention), six months and 12 months.

#### Primary outcome

##### Self-reported functional disability

The Modified Oswestry Low Back Pain Disability Questionnaire (ODQ), a valid and reliable measure of pain and physical function in LBP patients, will be used as the primary outcome measure [35]. Ten questions, each with six options, assess an individuals’ perceived activity restrictions in daily tasks. A minimum score of zero and a maximum score of five is allocated to each question and summed. The recent UK *feasibility* study (n = 57) of pedometer-driven walking in people with CLBP demonstrated a 6 to 7 point between group difference at six months and will be used as a comparator in this study [21].

#### Secondary outcomes

##### Physical activity level (PA)

Participants will complete the International Physical Activity Questionnaire short form (IPAQ) at each time point. The IPAQ is a well-established tool for assessing PA and is considered valid and reliable for assessing PA levels in clinical populations [36-39]. The IPAQ uses seven questions asking participants about the time they spent being physically active in the last seven days.

##### Participant beliefs/perceptions

The Fear-Avoidance Beliefs Questionnaire (FABQ) will be used to assess participants’ perceptions about how PA and work affects their CLBP [40]. The FABQ is a 16 item self-report questionnaire divided into two sub-scales with established reliability and validity in individuals with persistent pain conditions; PA (five questions) and work (11 questions). Participants rate each question on a scale from zero to six, were zero indicates complete disagreement with the statement and six indicates complete agreement. Four scores from the PA sub-scale and seven from the work sub-scale are summed giving maximum scores of 24 and 42 respectively.

Individuals’ beliefs regarding their future living with CLBP will be assessed using the Back Beliefs Questionnaire (BBQ). Individuals rate their level of agreement with 14 statements on a scale from one to five were one indicates complete disagreement and five indicates complete agreement. Five statements are considered ‘distractors’ and not used in scoring. Each statement score is reversed so that low scores represent negative beliefs and then summed to determine the final score. The BBQ has been found to have good validity (Cronbach’s Alpha = 0.70 to 0.75) and reliability (Intraclass Correlation Coefficient = 0.87) [41,42].

At baseline, participants will be asked to rate their ability to be more physically active compared to the previous week using the Global Rating of Change for Physical Activity questionnaire [43,44]. Participant ratings will be recorded as no change, worse, or better. If they answer worse or better, they will be asked to quantify the amount using one of the following markers: a tiny bit - almost the same; a little bit; somewhat; moderately; quite a bit, a great deal or a very great deal. Participants will also be asked to rate how important this change (or lack of change) is to them using the same markers. At each follow up assessment, participants will be asked to give a rating compared to the last time point.

##### Quality of life

Back pain significantly impacts quality of life [45]. Our study will use Quality Adjusted Life Years (QALYs) to estimate the impact of a walking programme on an individual’s quality of life. The EuroQol health survey instrument (EQ-5D-5 L) [46] is a self-administered survey tool that captures general health status. Participants rate their health on the day of testing over five domains (mobility, self-care, usual activities, pain/discomfort, and anxiety/depression) on a five-point scale and then score on a scale of 0 to 100 how their health today compares to their best (100) or worst (zero) health ‘imaginable’. An individual’s rating of each of the five health-related domains is summarised as a single score in the form of a utility estimate. The utility estimate will then be converted into a QALY for each participant and an average QALY for each group. We will compare the difference in QALYs at three, six, and 12 months.

##### Cost assessment

Direct and indirect healthcare-related costs associated with both intervention groups will be collected using a combination of patient diaries and questionnaires at each testing session throughout the 12 month study period. At baseline and each follow up visit, information will be collected regarding healthcare utilisation over the preceding 3-month period, including physiotherapy, family physician, and walk-in clinic visits, emergency room visits, and hospitalisations. Work status, work efficiency, time lost from unpaid work and absenteeism will be measured in each group using the Work Productivity and Activity Impairment questionnaire [47]. The difference in total costs will be compared at three, six, and 12 months.

### Randomisation

A computer generated random sequence will be performed using minimisation allocation with a 1:2 ratio to the back care advice and education group or back care advice and education plus pedometer-driven walking group [48]. To ensure adequate access to pedometers, randomised sequences will be created in blocks of 30 at a time (20 treatment: 10 control) and each allocation placed in a sealed blank envelop. As the self-management nature precludes double blinding, only the assessor for outcome measurements will be blinded to group allocation.

### Intervention

#### Back care advice and education

All participants will be given education and advice regarding self-management and the benefits of staying active [49]. Advice and education will take place in a single session, on a one to one basis for approximately one hour, by the research physiotherapist. Education and advice will be based on ‘The Back Book’ [50,51] which encourages a graded return to normal activities, addresses the nature of LBP, corrects unhelpful beliefs, and emphasises the need to use prophylactic pain control medication to allow activity [52,53]. The research physiotherapist will personalise the information using prior knowledge of the participant gained from clinical examination, baseline outcome measures and the pedometer trial results. Immediately following the back care advice and education session participants will be randomised into either the back care advice and education group or the back care advice and education plus pedometer-driven walking group.

#### Pedometer-driven walking programme

Participants allocated to the back care advice and education plus pedometer-driven walking group will undertake a physiotherapist-facilitated walking programme [21,54]. Each participant will be asked to wear a Yamax DigiWalker CW-701™ pedometer daily during the 12-week intervention period and record their daily steps in a walking diary. The aim of the intervention is to increase participants average daily step count. The walking programme is a behaviour change intervention in which the pedometer is used as a tool to help participants monitor their activity levels. The physiotherapist facilitates the process through the use of cognitive and behavioural techniques such as person-centred discussion of current activity behaviours, self-efficacy and barriers to increasing activity [55].

At week one, participants will undergo a 10 minute “self-efficacy walk” whilst wearing the pedometer [21]. The supervised self-efficacy walk assists participants in setting a walking goal (daily step count) they are confident in achieving over the following week. Participants return to see the physiotherapist at the end of week one to discuss any issues with the programme, pedometer or recording of desired information. A step target for week two will be agreed between the physiotherapist and the participant by referring to the mean daily step count recorded at baseline, the self-efficacy walk, and the average step count calculated from the walking diary. For weeks three to 12, the physiotherapist will phone each participant at a prearranged time, each week, to discuss his/her progress, document mean daily step count (recorded in diaries, and in the pedometer’s seven day memory) for the previous week, and agree to a new daily step target for the subsequent week. Throughout the walking intervention the physiotherapist will encourage participants towards self-management in goal setting, identifying suitable times and places for walking, dealing with any problems or difficulties that arise and refer to advice contained in the Back Book as appropriate. In this way the walking programme will be tailored to the individual.

Mean steps per day, for each week of the study will be calculated from participants’ daily records and will be used as both raw data and as change scores (numerical and percentage) recorded over the 12-week intervention period. Adherence to the walking group will be measured from participant reports of pedometer-derived data and calculated as a percentage of the prescribed number of steps completed [21]. Self-confidence and the belief that one can increase their PA through walking will be guided through the use of a five point self-efficacy scale developed by Marcus et al. [56]. High self-efficacy is related to reduced disability and the successful management of chronic conditions [57].

##### Focus groups

On completion of the 12-week walking programme, participants will be invited to participate in a focus group discussion designed to explore their thoughts and experiences of using pedometer-driven walking as a management tool for CLBP. Focus groups, led by an experienced moderator, will be established following every 30 participants who complete the walking programme. We anticipate recruiting five to six focus groups of six to eight people over the period of the study. One focus group will specifically recruit rural farm participants; conducted to address the feasibility of walking programmes in this setting. A checklist will be used to facilitate discussion on specific topics of interest to the researchers, yet at the same time allow for exploration of new topics or areas of interest to arise.

### Sample size

Given the non-invasive nature of a walking intervention likely carrying minimal health risk, we have chosen to use a 1:2 allocation ratio towards the walking intervention in order to increase confidence in interpreting or accepting any observed effect [58,59]. Results from the feasibility study informed initial calculations of sample size based on between group differences of 6–7 points in the ODQ. Given the wide confidence intervals expressed in these between group feasibility data and consdiering the recommended minimum important change of 10–12 points for individual improvement [60], we chose to first explore for an eight point between group difference. An 80% power calculation (alpha = 0.05), resulted in a minimum requirement of 174 participants (116 walking group, 58 standardised group). We anticipate a 15% loss to follow-up and will thus, aim to recruit 200 participants.

### Statistical analysis

Multiple regression and/or mixed model analyses will be used to determine predictive models that best test for differences and changes in both outcomes and costs. Both within- and between group models will be used in order to determine whether use of a pedometer in the manner described in this study is a predictor of these outcomes. The change in ODQ from baseline will be the main dependent variable. Other outcome measures will be entered as alternate dependent variables to determine whether they significantly alter or provide a stronger model. Personal, occupational, seasonal, clinical and anthropometric variables will be entered into the model in a stepwise manner to seek the models that best explain changes in outcomes. Descriptive and statistical results for personal, demographic, anthropometric and outcome measures will be presented in tabular and graphic format.

Focus group discussions will be audio-recorded, transcribed and imported into NVivo 10 (QSR International) software for analysis. Thematic analysis using inductive methods will be used to identify core ideas important to participants’ experience, meaning and reality of pedometer-driven walking [61].

### Training requirements

A physiotherapist will be specifically recruited to undertake the clinical examination screening of potential participants, provide the personalised back care advice and education to all participants, and facilitate the 12-week pedometer-driven walking group. Training, adapted from the work of McDonough et al. [21], will be provided prior to study commencement. Training will cover screening procedures, back care advice and education guidelines, pedometer-driven walking programme delivery and required documentation. Cognitive and behavioural approaches to managing people with chronic pain and promoting health behaviour change using techniques such as motivational interviewing, action and coping planning will underpin the training process [55].

Two blinded assessors will be responsible for collecting outcome data at baseline, three, six and 12 months. A standardised procedure will be developed by the research team to ensure a consistent assessment approach.

### Treatment fidelity

Random observation by a research team member of each face-to-face session between participant and physiotherapist will be carried out throughout the study intervention period. Checklists for each stage (screening examination, pedometer trial, back care advice and education session, walking programme delivery at week 1 & 2) will be established prior to recruitment and used to ensure treatment fidelity. Phone monitoring of participants will be recorded weekly and subject to random inspections. Any discrepancies or concerns will be addressed through discussion.

Evaluating participants’ adherence and performance with the pedometer-driven walking programme will be achieved through weekly phone monitoring and self-report of daily step count recorded in a dairy. All participant contact times will be recorded by the physiotherapist.

### Adverse events

Minimal adverse events are anticipated [21]. Any untoward or undesired experiences associated with the pedometer-driven walking programme will be recorded.

## Discussion

Although walking is recognised as a fundamental human activity, it is under-utilised in modern society and there has been little focus on its use as an intervention strategy for CLBP. The study outlined in this paper has been designed to determine the effectiveness of a community-based pedometer-driven walking programme as a low cost, easily accessible, and sustainable means of physical activity to improve disability and clinical outcomes for people with CLBP in a Saskatchewan context. The study design builds on the knowledge gained in recent reviews [14,19,22,62], prior protocol development [20,63] and recent walking studies [21,64]. Results from this study will inform future exercise-based strategies for larger comparative international clinical trials aimed at reducing disability in those suffering from CLBP as well as establishing the feasibility of pedometer-driven walking programmes in the farming population.
